# Results of screw fixation in Mason type II radial head fractures

**DOI:** 10.1186/s40064-016-2189-2

**Published:** 2016-04-27

**Authors:** Murat Demiroglu, Kahraman Ozturk, Mehmet Baydar, Omer F. Kumbuloglu, Ayse Sencan, Serkan Aykut, Bulent Kilic

**Affiliations:** Department of Orthopaedics, Göztepe Training and Research Hospital, Medeniyet University, Dr. Erkin Cad. Kadıkoy, Istanbul, Turkey; Hand Surgery Department, MS Baltalimani Training and Research Hospital, Istanbul, Turkey; Hand Surgery, Bagcilar Training and Research Hospital, Istanbul, Turkey; Gelisim University Health Sciences, Istanbul, Turkey

**Keywords:** Radial head, Fractures, Mason classification, ORIF, Screw fixation, Outcome

## Abstract

**Purpose:**

The treatment of Mason type II fractures is controversial, and the aim of our study is to define the outcome of surgical treatment with screw fixation in the Mason type II radial head fracture.

**Methods:**

The study was carried out between 2011 and 2015, and included 14 men and 9 women, with isolated Mason type II radial head fractures which were treated operatively with screw fixation. Cases involving the additional ligament injury or fractures in other areas, or having a follow-up period which is greater than 11 months were excluded. The clinical and radiological results of our patients were assessed, using the Mayo Elbow Performance Score (MEPS).

**Results:**

The average MEPS was 95.86 points. 100 degree arcs of motion were attained by a total of 21 patients (91 %) for both flexion–extension and pronation–supination. Nevertheless, 2 patients (9 %) did not recover the 100 degree arcs for the flexion–extension.

**Conclusion:**

Anatomical reduction of type II radial head fractures through open surgery and fixation with screws can have favorable results.

**Level of evidence:**

Level IV, Retrospective design.

## Background

Fractures of the radial head are relatively common. Overall, in both children and adults, they represent approximately 5.4 % of all fractures, and 33 % of elbow fractures (Mason 1954).

The mechanism of injury is usually a fall on an outstretched arm, and, in rare cases, direct trauma (Morrey 2000; Mason 1954; Johnston 1962). These fractures are typically seen in isolation, but they may be accompanied by other fractures, dislocations, or soft tissue injuries.

The classification of fractures of radial head and neck was first done by Mason (1954), and later modified by Johnston (1962). While conservative methods are primarily used to treat Mason I fractures, the treatment of Mason II fractures is controversial (Yoon and Athwal 2012). Mason III (comminuted) fractures, when technically possible, are treated primarily through open reduction and internal fixation, via the use of screws or plates (Sanders and French 1986; Yoon and Athwal 2012). Although the treatment options for type I and type III fractures are well-defined, there is no agreed method in treating type II fractures. This study aims at evaluating the effectiveness of treating Mason II radial head fractures through the fixation with micro acutrack 2 screw (Acumed) in a retrospective series of 23 patients.

## Methods

This is a retrospective study done in a single tertiary trauma center by two experienced trauma surgeons between 2011 and 2015. Of the eighty-three (83) patients with radial head fractures, twenty-eight (28) were diagnosed with type II radial head fractures. One of the patients refused to undergo surgery and two had a medial collateral ligament (MCL) injury. Moreover, two other patients were excluded from this study, because their follow-ups were done at another hospital. The study was carried out and completed with a group of 23 patients (14 men and 9 women).

The patients who were evaluated and reported in this study met with the following criteria: (1) the patient had to give permission to be included in the study, (2) the patient had to have a Mason II fracture (the radial head intra-articular should be in one piece and displacement should be at least 2 mm), (3) the follow-up period of the patient should be longer than 11 months, (4) the patient must not have another osseous and ligamentous pathology, and (5) surgical treatment of the patient had be applied as osteosynthesis via the use of screws.

Cases involving the additional ligament injury or fractures in other areas, or having a follow-up period which is less than 11 months, were excluded.

The amount of slip at the articular surface of the radial head was determined through computerized tomography (CT). The average wait time for surgery was 2 days (range 0–5 days). Radiological and functional evaluations were assessed for 23 patients with radial head fractures, which were all closed injuries, at an average of 26 months postoperatively. These fractures existed in the dominant arm for 14 patients, and in the non-dominant arm for 9 patients. In this study, right and left sides were affected by the radial head fractures in 13 and 10 patients respectively (Table 1). The average age of the patients with surgery was 35 years (range 24–53 years). Injuries of 22 patients were due to a simple fall; 1 was due to a bicycle accident.

**Table 1:** Patients’ data with Mason II radial head fractures

Case number	Sex (M/F)	Side	Dominant (±)	MEPS (Pt)	QuickDash (Pt)	Followup (month)	VAS (0 → 10)	Supi/Pron (°)	Fleks/Ekst	ROM (°)
1	F	R	+	95	2.3	40	0	70/70	120/10	110
2	M	L	−	100	0	44	0	70/60	110/10	100
3	M	L	−	90	9.1	32	0	80/60	120/20	100
4	M	R	+	90	2.3	24	1	80/60	120/10	110
5	F	R	+	95	0	18	1	70/60	110/10	100
6	F	R	+	95	0	26	0	80/60	100/10	90
7	F	L	+	100	0	20	0	70/60	110/10	100
8	M	L	−	95	2.3	22	0	80/70	130/0	130
9	F	L	−	100	0	38	0	80/70	120/0	120
10	F	R	+	95	0	36	1	70/70	120/0	120
11	M	R	+	95	0	32	0	70/60	130/10	120
12	M	L	−	95	0	30	0	80/60	130/10	120
13	M	R	+	100	0	22	1	80/60	120/20	100
14	M	R	+	95	6.8	24	0	70/60	130/0	130
15	F	R	+	95	0	18	0	80/70	130/10	120
16	M	L	−	95	0	20	0	70/60	110/10	100
17	M	L	−	100	0	20	1	70/60	120/0	120
18	M	R	+	95	0	22	1	70/60	120/0	120
19	F	R	+	100	0	30	0	70/70	130/0	130
20	M	R	+	95	2.3	26	0	70/60	130/10	120
21	M	R	+	100	0	20	0	70/60	120/10	110
22	M	L	−	95	4.5	28	0	70/70	120/10	110
23	F	L	−	90	2.3	24	0	60/50	120/20	90

All Mason II fractures were fixed by the use of screws only. Ultrasound-Guided Infraclavicular Brachial plexus block anesthesia was administered to all the patients for the surgery. 1 g of cefazolin IV was administered to all of the patients, 30 min before the surgery, as antibiotic prophylaxis. Pneumatic tourniquet with 250 mmHg pressure was used in all operations, which were performed on a hand table.

The Extended Extensor Digitorum Communis (EDC) splitting approach, which involved detaching the anterior half of the EDC, as well as Extensor Carpi Radialis Brevis (ECRB) from the lateral epicondyle, was used for the exposure.

**Fig. 1:**
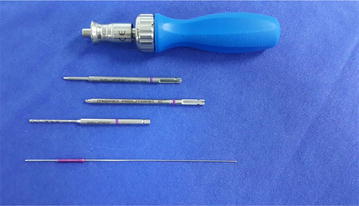
Acutrak 2 (TM)—Micro installation tool

Reduction of articular step in the radial head, which was achieved during surgery without the use of grafts (Fig. 1).

**Fig. 2:**
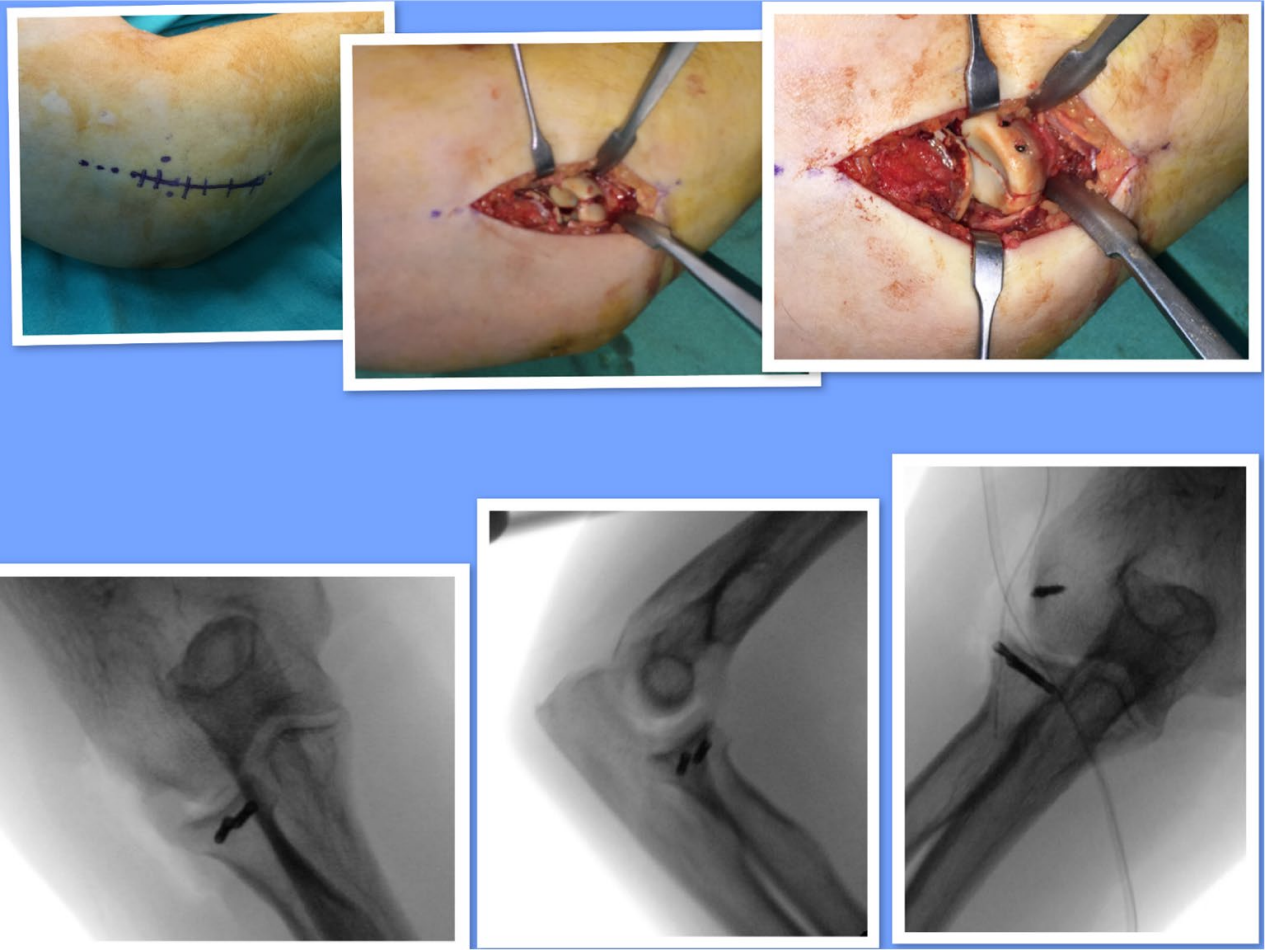
Kocher approach; minimal displacement radial head fracture; after reduction, fracture was fixated with 2 acutrak 2-micro screw. Screw position was controlled by the C arm scopy; LCL was repaired with anchor

Following the anatomical repositioning of the fracture fragment, two Acutrak (Acumed) 2 TM microcannulated compressive headless screws, generally 20 mm in length, were used (Fig. 1). The position and length of the screws were checked, along with forearm rotation through fluoroscopic control (Fig. 2). The annular ligament was repaired after fixing the radial head. Bleeding was controlled after deflating the tourniquet in each surgery. After the operation, a long-arm splint was applied, in forearm neutral rotation, to the patients, and used for 3 days.

Active and active-assisted physiotherapy were initiated on the second or third day after surgery for the recovery of full ROM as a home-based rehabilitation. Varus, valgus stresses, and resistive exercises were banned for 6 weeks. If LCL was repaired, forearm rotation exercises was performed in 90 degree of elbow flexion, and extension exercises was performed only in the pronation position of the forearm for 3 weeks.

Clinical and radiological examinations of all the patients were done by two orthopaedic surgeons (SA, MB, AS).

At clinical assessment, there were no patients with a history of restricted movement or pain prior to the fracture. The range of motion was assessed on the 15th day, and the patients with restricted range of motion were prescribed physical therapy under the supervision of physiotherapists with continuos passive range of motion device. MEPS and Quick Dash, VAS ve and ROM assessments were made during the 6th and 11th months.

Antero-posterior and lateral radiographies of the elbow were checked for all the patients, in order to verify the correct setting of the implant, heterotopic ossification, and ulno-humeral osteoarthrosis.

Roentgenographic controls were performed after osteosynthesis on the 2nd day, and in the 2nd and 6th month.

The criteria of MEPS, The Disabilities of the Arm, Shoulder and Hand Score (QuickDash), Visual Analog Score (VAS), and Morrey’s functional arc were all assessed for patients, in order to evaluate the functional results of recovery. Patients stayed in hospital between 1 and 3 days (average 1.4 days), and the average follow-up time was 26 months. A goniometer was used to measure the elbow ROM in flexion–extension and pronation–supination. Patients were divided into three groups, based on the recovered ROM:

1. patients who had recovered at least a 100 degree arc in both flexion–extension and pronation–supination,
2. patients who had recovered at least a 100 degree arc only in flexion–extension,
3. patients who had recovered at least 100 degree, but in none of them. Also, there were patients who had less than a 100 degree arc.

A ROM greater than 100 degrees, as in Morrey’s definition of the functional arc, was necessary to perform most of the activities of daily living (Morrey et al. 1981). Elbow stability was evaluated for all patients by using the varus-valgus stress and the lateral pivot shift tests, as described by O’Driscoll for postero-lateral rotatory instability (Ertürer et al. 2010). Additionally, wrist motion was evaluated by considering the presence of pain.

The overall satisfaction with the elbow pain was ranked on a scale between 0 (no pain) to 10 (maximum pain), according to VAS, based on patients’ reports (Table 1; Zarratini et al. 2012). MEPS was used to assess elbow performance in terms of pain, arc of motion, stability, and the ability required to perform daily activities, along with QuickDASH score, which consists of 11 questions that are elbow-related (Morrey et al. 1993; Hudak et al. 1996; Duckworth and Clement 2012).

### Statistical analysis

The 2007 Number Cruncher Statistical System (NCSS; Kaysville, Utah, USA) statistical software was used for statistical analysis. A Wilcoxon signed-rank test was used to assess the descriptive statistical methods (mean, frequency, ratio, minimum, and maximum), as well as the measurements of variables without a normal distribution, after the treatment.

## Clinical results

Our aim was to reach a pain-free flexion–extension and pronation–supination arc of greater than 100 degrees in the joint. All patients were satisfied with the procedures, and except for two patients, the range of motion exceeded 100 degrees. The post-operative range of motion (ROM), which was greater than 100 degree for almost all patients in both flexion–extension and pronation supination, was established without any complications. All elbows were stable.

The average flexion–extension range of motion and loss of movement, which were 120 (range 90–130) and 8.26 (range 0–20) degree respectively, resulted in a supination–pronation range of motion for the elbows of all patients, with the average being 111 (range 90–130).

Seventeen patients (73 %) suffered from no pain, while six patients (27 %) had mild pain when engaging in demanding activities.

One patient (4 %) had clicking sensations in the elbow.

No patient complained about the weakness or instability of elbow on clinical testing.

The average MEPS after surgery was 95.86 points. According to the MEPS, 23 ‘‘excellent’’ results (100 %) were achieved.

The average DASH-score was 1.38 points (range 0–9.1 points).

**Fig. 3:**
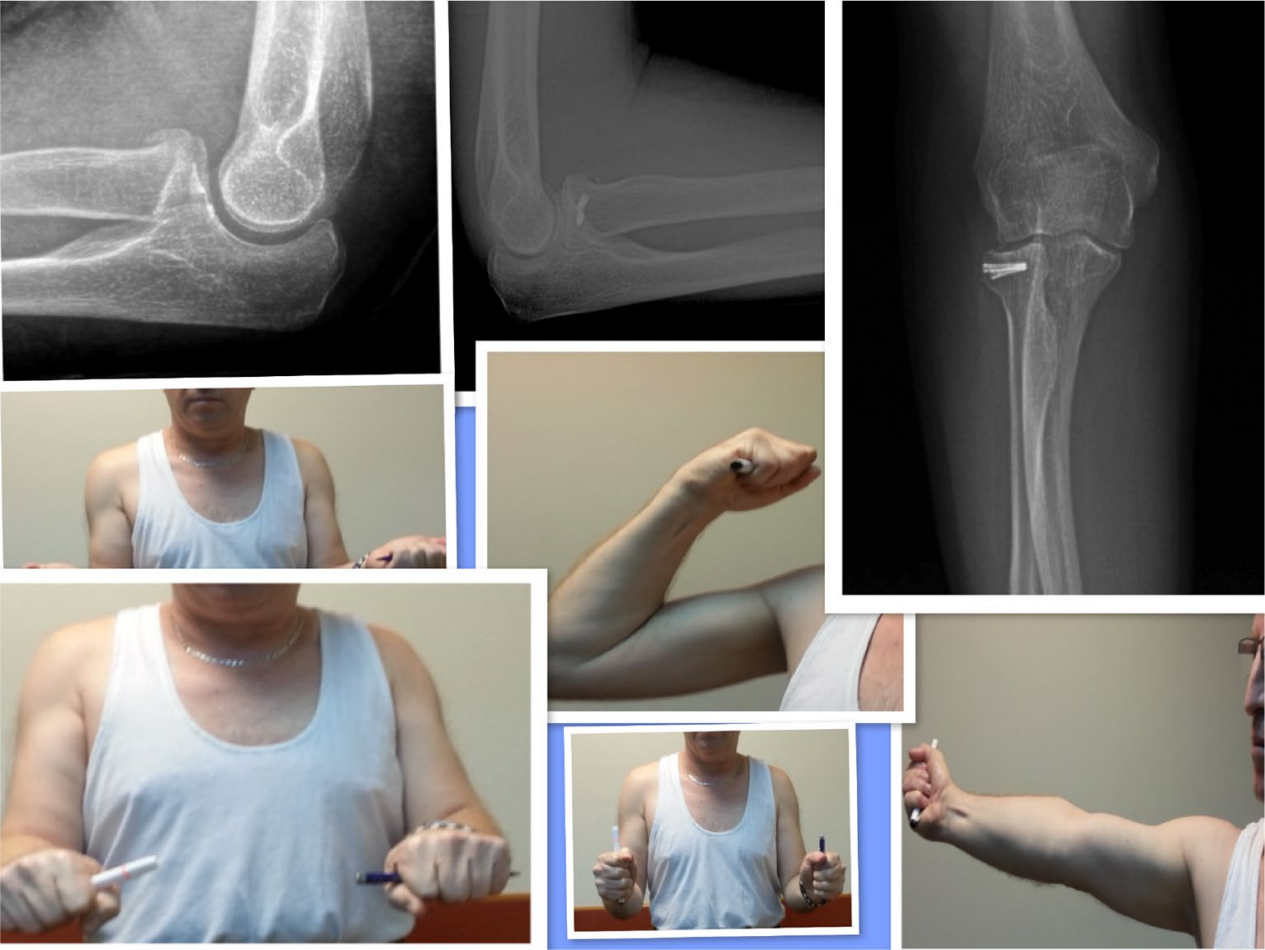
50-year old patient. Good to excellent results

**Fig. 4:**
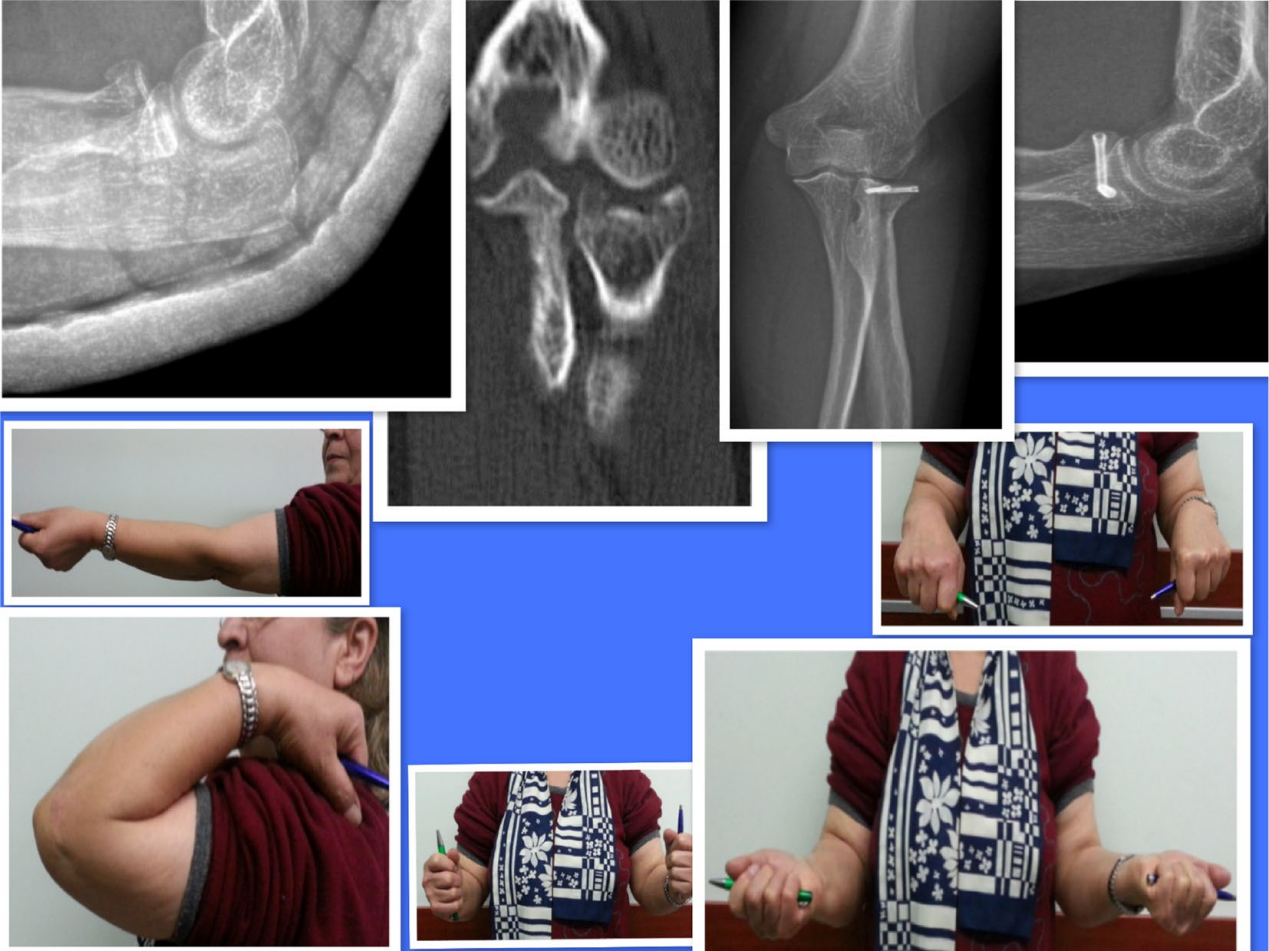
52-year old female. Good to excellent results

A total of 21 patients (91 %) recovered their flexion–extension and pronation–supination. Two patients (9 %) did not recover their flexion–extension arc but recovered pronation–supination arc without complaints and pain (Figs. 3, 4).

Stability of screw fixation was preserved for all the patients and neurological complications or infection were not encountered.

## Discussion

Radial head fractures are the most frequently encountered fractures in the elbow (Mason 1954; Morrey et al. 1993). The results obtained via a conservative treatment may be satisfactory if the fracture is not displaced, or is minimally displaced but movement is not impeded (Duckworth and Clement 2012).

The radial head is recognised as an important stabilizer of the elbow; it has primary importance in the absence of medial collateral ligament. Displaced radial head fractures can be isolated, or can exist with soft tissue damage and other regional bone fractures.

In 1954, when CT evaluation did not exist, Mason classified these fractures and Johnston modified them in 1962. It is this classification, along with the later versions, that has become popular (Mason 1954; Johnston 1962).

Shulman does not recommend surgical treatment in the fractures with displacements of less than 2 mm (Shulman et al. 2015).

Mason type II fractures are two part fractures of the radial head with displacement. The optimal treatment for Mason II fractures of radial head is still controversial. Satisfactory results have been reported in literature, with both open reduction (Esser et al. 1995; Khalfayan et al. 1992; King et al. 1991; Pearce and Gallannaugh 1996; Van Glabbeek et al. 2001) and conservative treatment (Weseley et al. 1983).

For the surgical exposure, Desloges and Louati (2014), suggested an extended EDC approach, because it can provide a wide-angle view in a radial head fracture surgery. This is the approach used in this study. However, the Kocher approach is preferable if LCL injury is suspected.

In the treatment of Mason II fracture, K-wire has been tested along with screws; however, we do not recommend using it because of the possibility of migration and failure to compress the fracture line (Ertürer et al. 2010). Two parallel screws were used in our fracture fixation and any fixation failure was not detected.

In the study done by Zarrattini et al. (2012), where twenty-four (24) and thirty-five (35) patients were treated via radial head excision and open reduction internal fixation (ORIF) respectively, it was reported that ORIF had an advantage over radial head excision, in terms of pain and the range of motion power recovery.

Another major study by Weseley et al. (1983), where all isolated radial head fractures of 387 patients were treated without exception for type conservative, it was concluded that perfect sound results could be obtained (Weseley et al. 1983). Although it does not correspond to general consents, Weseley et al. (1983) emphasized that it is crucial to convince the patients of the importance of early movement.

The most common problem encountered during surgical or conservative treatment of Mason type II fractures is eburnation. This was not a problem in the series of this study, except for two (2) patients who had a flexion–extension with a degree of less than 100.

Charalambous et al. (2006), who conducted cadaveric studies, found that the fixation of fractures is superior to excision or replacement.

Early movement in radial head fractures forms the basis of the factors affecting rehabilitation and outcome of the treatment. In this study, fixation using two screws eliminated the concern over early movement.

According to Longo et al. (2008), there are many evaluation systems for the outcome of the treatment of elbows. Quick DASH is the shortened version of DASH. A final score of zero (0) indicates the absence of disability, while one of one hundred (100) is an indication of the likelihood of severe disability. MEPS is one of the most commonly used evaluation methods, and the points between 90 and 100 indicate perfect results (Hudak et al. 1996; Duckworth and Clement 2012).

Esser et al. (1995) reported sound and perfect results after surgery for a mixed series of people having Mason II fractures. This is in agreement with our study.

Khalfayan et al. (1992) found out that the patients who did not have a surgery, had more pain along with functional limitation, hence, surgery was suggested, and this was compatible with our study.

Solarino et al. (2015) recommended using radial head excision in the cases where the patient is over 65, and there is no other elbow ligament injury.

King et al. (1991) obtained satisfactory functional result with an average of 142 degrees of motion after surgery. Similarly, Pearce and Gallannaugh (1996), who studied similar fractures via the use of Herbert screws, reported good-perfect results and suggested surgery. In the study of Van Glabbeek et al. (2001), surgical treatment was also suggested for Mason II fractures.

Burkhart et al. (2015) recommend surgery in these types of fractures, even if there are other good treatments available, to avoid the possibility of development of post-traumatic arthritis, due to the intra-articular fracture.

In a biomechanical study done by 15, it was demonstrated that the flexion–extension of 100 degrees and pronation–supination with 100 degrees are sufficient for daily activities (Morrey et al. 1981). In our study, these criteria were taken into account, and only two (2) patients had disability of flexion–extension, albeit at 100 degrees.

Finally, according to Yoon and Athwal (2012), ORIF is a good alternative for obtaining successful results for radial head fractures showing displacement.

Conservative treatment has been advocated for the minimally displaced Mason type II fractures, but the success rate decreases as the displacement increases. Moreover, it is not always easy to determine the degree of displacement clearly, or the mild mechanical block during motion in the acute phase of the trauma.

Also, there are also no clear-cut off value for the displacement amount to decide whether to treat these fractures conservatively or not.

So, we usually recommend surgical treatment of Mason type II radial head fractures, and believe in the importance of anatomical reduction with early movement, which usually ends with a predictive and successful outcome in this cohort of young and middle-aged adults.

## Conclusion

The limiting factors in our study are the relatively few patients who were included, and its lack of a control group, which is a limitation to our study. Nevertheless, the results show that Acutrak 2 micro screw fixation for Mason type II fracture is a suitable option with predictive results for acute treatment of Mason type II radial head fractures.
